# Baicalein as a potent antifungal agent against *Candida albicans*: synergy with fluconazole and sustainable production through probiotic-mediated bioconversion

**DOI:** 10.3389/fmicb.2025.1562103

**Published:** 2025-02-25

**Authors:** Hang Zhai, Jialing Zeng, Xiaonan Ma, Fuju Wang, Wei Xu, Ming Song, Weizhuo Xu

**Affiliations:** ^1^School of Life Science and Biopharmaceutics, Shenyang Pharmaceutical University, Benxi, China; ^2^Global Biologicals Inc., Beijing, China; ^3^Faculty of Functional Foods and Wine, Shenyang Pharmaceutical University, Benxi, China

**Keywords:** antifungal, baicalein, baicalin, *Lactobacillus rhamnosus*, biotransformation, β-glucuronidase

## Abstract

Fungal infections, particularly those caused by *Candida albicans*, represent a significant global health concern, with drug resistance and biofilm formation posing considerable challenges to effective treatment. Baicalein, a flavonoid derived from baicalin found in *Scutellaria baicalensis*, has demonstrated considerable antifungal efficacy. Moreover, the combination of baicalein and fluconazole demonstrated a notable synergistic effect. Given the restricted natural accessibility of baicalein, *Lactobacillus rhamnosus* has been identified as a microbial agent capable of converting baicalin to baicalein via whole-cell catalysis. This process has been shown to achieve a molar yield of 66% at a substrate concentration of 4 g/L under optimized conditions. In addition to the antifungal properties of baicalein, *L. rhamnosus* demonstrated intrinsic antifungal activity against *C. albicans*. The combination of baicalein and *L. rhamnosus* resulted in a notable enhancement in the inhibition of *C. albicans* growth. The key enzyme responsible for baicalin hydrolysis, *β*-glucuronidase (*Lr*GUS2), is indicative of the biotransformation potential of *L. rhamnosus*. This study demonstrates the potent antifungal activity of baicalein, its synergistic effects with fluconazole, and the ability of *L. rhamnosus* to efficiently convert baicalin into baicalein. These findings highlight the potential for developing baicalein as a novel antifungal agent, particularly in combination therapies for resistant *C. albicans* infections, and provide a scalable, safe method for baicalein production using probiotics.

## 1 Introduction

Fungal infections have emerged as a significant public health challenge worldwide, with approximately 1 billion individuals affected by superficial infections involving the skin, nails, or hair. Millions more suffer from mucosal fungal infections, and invasive fungal infections are responsible for an average of over 1.5 million deaths annually, underscoring the severe threat they pose to human health globally (Bongomin et al., 2017; Bajpai et al., 2019). Among the most prevalent fungal pathogens is *C. albicans*, a common component of the human microbiota that typically colonizes the gastrointestinal tract, oral cavity, and skin. However, immune compromise or dysbiosis within the microbiota can result in overgrowth and biofilm formation, leading to *Candida*-associated infections (Kaur and Nobile, 2023), which are implicated in conditions such as inflammatory bowel disease (IBD).

Three primary classes of antifungal drugs are currently used in clinical practice: imidazoles, polyenes, and echinocandins. These agents target distinct fungal pathways by inhibiting cytochrome P450 (CYP51), ergosterol synthesis in the fungal cell membrane, and (1,3)-*β*-D-glucan synthase, respectively (Larru and Zaoutis, 2013; Perfect, 2017). Unfortunately, the widespread development of drug-resistant fungal strains has limited the effectiveness of these therapies, necessitating the urgent development of new antifungal agents that combine high efficacy with low toxicity.

Recent antifungal agents entering clinical trials are largely small molecules. For example, structural optimization of azoles targeting CYP51 has yielded compounds like VT1129 and VT1161, which exhibit over 1,000-fold greater binding affinity (Warrilow et al., 2014; Break et al., 2018). In addition, novel agents have emerged from natural product screenings, such as SCY-078, a potent *β*-1,3-D-glucan synthase inhibitor (Peláez et al., 2000), F901318, which impairs pyrimidine biosynthesis via dihydroorotate dehydrogenase (DHODH) inhibition (Campoy and Adrio, 2017), and APX001A (E1210), which disrupts glycosylphosphatidylinositol (GPI) synthesis by inhibiting inositol acylation of glucosamine-PI (Watanabe et al., 2012). Beyond small molecules, probiotics represent a promising alternative for managing fungal dysbiosis and preventing opportunistic fungal colonization (Kunyeit et al., 2023). Notably, *Lactobacillus paracasei* has demonstrated both bacteriostatic and bactericidal activity against *C. albicans* and *Saccharomyces cerevisiae* (Atanassova et al., 2003). Moreover, a combination gel containing *Lactobacillus rhamnosus* GG, *Lactobacillus pentosus* KCA1, and *Lactobacillus plantarum* WCFS1 has shown comparable efficacy to fluconazole in treating vulvovaginal candidosis (VVC), suggesting that probiotics may offer a new opportunity for antifungal therapy (Oerlemans et al., 2020).

A screening of 1,266 compounds with known pharmaceutical activity identified Enoxacin, a fluoroquinolone, as a promising antifungal agent. Enoxacin prolonged the survival of *C. albicans*-infected nematodes and inhibited fungal filamentation *in vivo* (Breger et al., 2007). This led us to explore the antifungal potential of flavonoids, given the structural similarity between Enoxacin and flavonoid scaffolds. Flavonoids extracted from *S. baicalensis* roots exhibit a broad range of biological activities, including antioxidant, anti-inflammatory, antiviral, and antithrombotic effects. Notably, baicalein and wogonin induce apoptosis-like programmed cell death via reactive oxygen species generation (Da et al., 2019), and baicalein exerts protective effects in mouse models of keratitis by downregulating the TSLP/TSLPR pathway (Zhu et al., 2021). Despite the promising bioactivity of baicalein, its aglycone content is notably low in *S. baicalensis* root extracts compared to its glycoside derivatives, such as baicalin (3.520 ~ 11.400%) and wogonoside (5.070 ~ 10.300%), with baicalein and wogonin content as low as 0.073 ~ 0.247% and 0.002 ~ 0.061%, respectively (Wang et al., 2007; Makino et al., 2008; Shen et al., 2019). The complexity and inefficiency of isolating and purifying baicalein have limited its therapeutic development.

In our study, it was first demonstrated that baicalein possesses stronger antifungal activity than baicalin. Furthermore, baicalein, when combined with fluconazole, exhibited synergistic effects against fluconazole-resistant *C. albicans* strains and efficiently inhibited the growth of resistant fungal hyphae. To overcome the challenge of baicalein isolation, biotransformation was employed as a green and efficient approach to convert baicalin into baicalein using the probiotic *L. rhamnosus*. The key enzyme, *β*-glucuronidase (*Lr*GUS2), responsible for this conversion was cloned and expressed, and its ability to hydrolyze baicalin into baicalein was confirmed. Optimized transformation conditions resulted in a 1.57- and 4.07-fold increase in baicalein yield, respectively. Given the antifungal potential of probiotics, the combined effects of *L. rhamnosus* and baicalein on *C. albicans* growth were investigated. The results demonstrated that the biotransformation solution significantly inhibited *C. albicans* growth, suggesting a novel approach for utilizing probiotics and baicalein as a combined antifungal therapy, particularly in the context of intestinal *C. albicans* infections.

## 2 Materials and methods

### 2.1 General experimental materials

Fluconazole and nystatin were purchased from the China National Institute for Food and Drug Control. Baicalin and baicalein were obtained from Jiaxing Sicheng Chemical Co., Ltd. (Jiangxi, China). Polyamide film thin-layer chromatography plates were purchased from Sijia Biochemical Plastic Factory (Luqiao Co. Beijing, China). Plasmid and genome extraction kits were provided by Tiangen Biochemical Technology Co., Ltd. (Beijing, China). *Escherichia coli* DH-5α, BL21 (DE3), and vectors pMD19-T (Simple) and pET-28a(+) were obtained from Takara (Japan). The sensitive strains *C. albicans* CMCC (F) 98,001, *Yarrowia lipolytica* CGMCC 2.1405, and *Yarrowia lipolytica* CGMCC 2.2087 were sourced from the China General Microbial Culture Collection Center. *Gibberella* sp. CICC 2498, *Cunninghamella echinulata* CGMCC 3.967, *Absidia coerulea* CICC 41050, *Cunninghamella blakesleeana* CGMCC 3.970, and *Cunninghamella elegans* CGMCC 3.910 were acquired from the China Industrial Microbial Culture Collection and Management Center. Other strains including *Aspergillus niger*, *Penicillium*, *Paecilomyces lilacinus*, *Saccharomyces cerevisiae*, *Lactobacillus rhamnosus*, *Lactobacillus paracasei*, *Lactobacillus gasseri*, and resistant *C. albicans* are laboratory preserved strains.

### 2.2 General experimental methods

The whole-cell biotransformation process of *L. rhamnosus*. MRS medium (20 g/L glucose, 10 g/L peptone, 4 g/L yeast extract powder, 8 g/L beef extract, 2 g/L triammonium citrate, 5 g/L sodium acetate, 2 g/L K_2_HPO_4_, 0.2 g/L MgSO_4_, 0.04 g/L MnSO_4_, 1 mL/L Tween 80, pH 6.5) was used as the seed medium and transformation medium for probiotics. When 2% (w/v) agar was added to it, it was referred to as MRS solid medium, which was used for the activation of probiotics. The *L. rhamnosus* was incubated on MRS solid medium at 37°C for 48 h. Following satisfactory growth, a loopful of culture was inoculated into a 250-mL Erlenmeyer flask containing 30 mL of MRS medium and incubated at 37°C with shaking at 200 rpm for 16 h. This seed culture was transferred into fresh 50 mL MRS medium at 5% (v/v), with baicalin added to a final concentration of 0.5 g/L, and transformed at 37°C and 200 rpm for an appropriate time. Samples (5 mL) were collected and mixed with an equal volume of methanol, sonicated for 15 min, and centrifuged at 8000 rpm for 5 min. The supernatant was analyzed via TLC and HPLC. TLC used polyamide as the filler, with toluene, ethyl acetate, methanol, and formic acid (10:3:1:2) as the developing solvent. After pre-saturation for 30 min, plates were developed for 45 min, dried, and observed under UV light at 254 nm. HPLC was performed using methanol, water, and phosphoric acid (47:53:0.2) as the mobile phase, with detection at 280 nm, a flow rate of 1 mL/min, and a column temperature of 40°C. The actual concentrations of baicalin and baicalein were calculated using standard curves, and the molar yield was calculated as the ratio of the molar concentration of baicalein produced to the molar concentration of baicalin added.

### 2.3 Biotransformation process

#### 2.3.1 Strain screening

Potato Dextrose Agar (PDA, 200 g/L potato, 20 g/L glucose, 20 g/L agar) medium was used for the activation of filamentous fungi, and fungal transformation medium (15 g/L glucose, 15 g/L sucrose, 5 g/L peptone, 0.5 g/L KCl, 0.01 g/L FeSO_4_⋅7H2O, 0.5 g/L MgSO_4_, pH 7.0) was used as the seed medium and transformation medium for filamentous fungi. Filamentous fungi was activated onto PDA medium at 28°C for 7 days. The spores were washed with 5 mL of fungal transformation medium and then added to a 250-mL Erlenmeyer flask containing 50 mL of fungal transformation medium. The flask was incubated at 28°C with shaking at 200 rpm for 3 days. This culture was transferred into fresh 50 mL fungal transformation medium at 5% (v/v), with baicalin added to a final concentration of 0.5 g/L, and transformed at 28°C for 5 days.

Yeast culture medium (20 g/L glucose, 10 g/L peptone, 5 g/L yeast extract powder, pH 7.0) was used as the seed medium for yeasts. When 2% (w/v) agar was added to it, it was referred to as yeast solid medium, which was used for the activation of yeasts. Yeast transformation medium (30 g/L sucrose, 15 g/L peptone, 12 g/L (NH_4_)_2_SO_4_, 5 g/L K_2_HPO_4_, 0.5 g/L MgSO4, pH 6.5) was used as the transformation medium for yeasts. Yeast strains were activated onto yeast solid medium at 28°C for 2 days. Sterile inoculation loop was used to scrape one loop of culture and then added to a 250-mL Erlenmeyer flask containing 50 mL of yeast culture medium. The flask was incubated at 28°C with shaking at 200 rpm for 1 days. This culture was transferred into fresh 50 mL yeast transformation medium at 5% (v/v), with baicalin added to a final concentration of 0.5 g/L, and transformed at 28°C for 3 days.

MRS medium was used as the seed medium and transformation medium for probiotics. When 2% (w/v) agar was added to it, it was referred to as MRS solid medium. Probiotics were activated onto MRS solid medium at 37°C for 2 days. Sterile inoculation loop was used to scrape one loop of culture and then added to a 250-mL Erlenmeyer flask containing 50 mL of MRS medium. The flask was incubated at 37°C with shaking at 200 rpm for 1 days. This culture was transferred into fresh 50 mL yeast transformation medium at 5% (v/v), with baicalin added to a final concentration of 0.5 g/L, and transformed at 37°C for 3 days.

Three control experiments were conducted: (1) Substrate control group (substrate added directly to the culture medium) to assess substrate stability, (2) Bacterial control group (without baicalin) to assess any interference from strain metabolites, and (3) Pure culture medium control to exclude medium component interference. TLC detection was performed according to Method 2.2, and HPLC analysis was also conducted.

#### 2.3.2 Optimization of transformation conditions

##### 2.3.2.1 Turbidity for determining growth curve

The *L. rhamnosus* was incubated on MRS solid medium at 37°C for 48 h. A loopful of culture was inoculated into 50 mL of MRS medium and shaken at 37°C for 72 h. Samples were taken at regular intervals, and absorbance values at OD_625_ and pH were measured to plot the growth curve.

##### 2.3.2.2 Transformation method

The method of growing cell transformation was the whole-cell biotransformation process of *L. rhamnosus* mentioned in Method 2.2. For resting cells transformation, the seed culture was centrifuged, washed with PBS buffer (10 mM, pH 7.4), and resuspended in 50 mL PBS buffer before adding baicalin to a final concentration of 0.5 g/L. The transformation was conducted at 37°C for 3 days. The remaining conditions were consistent with the growing cell transformation.

##### 2.3.2.3 Optimization of transformation conditions

The effects of various carbon sources (soluble starch, glucose, maltose, lactose, galactose, and sucrose) on the molar yield of baicalein were investigated in MRS medium according to Method 2.2, and the effects of the concentration of the carbon sources were investigated after screening to the optimum carbon source. The effects of various nitrogen sources (ammonium sulfate, soybean cake powder, peptone, pancreatic peptone, soybean peptone, and yeast powder) on the molar yield of baicalein were investigated, and the effects of the concentration of the nitrogen sources were investigated after screening to the optimum nitrogen source. The filling volume of shake flask can affect the dissolved oxygen, which in turn affects the growth of the *L. rhamnosus*. Therefore, we investigated the effect of different filling volumes (20 mL to 150 mL) in 250-mL shake flasks on the molar yield of baicalein. Similarly, the inoculation percentage was also investigated using the same method, with the ratio ranging from 2 to 16% (v/v). pH not only affects the growth state of the microorganisms but also has a significant impact on enzyme activity. An appropriate pH is crucial for the conversion of baicalein. We investigated the effects of five different initial pH values (4.5, 5.5, 6.5, 7.5, and 8.5) on the conversion of baicalein. Due to the relatively low solubility of baicalin in water, which reduces the conversion rate, we compared the effects of different solvents on the conversion of baicalin. These solvents included water, DMF (dimethylformamide), DMSO (dimethyl sulfoxide), and acetate buffer (pH 5.0).

### 2.4 Cloning and expression of key enzymes

*Lacticaseibacillus rhamnosus* genomic DNA was extracted using a genome extraction kit [Tiangen Biochemical Technology Co., Ltd. (Beijing, China)]. The *uidA* gene was amplified by PCR with primers (5′-GGAATTCGGCTTTTTGCATGGAGAC-3′ and 5′-CCCAAGCTTCACCCAAAACTTACTTTGC-3′), pre-denatured at 95°C for 5 min, followed by 30 cycles of denaturation at 95°C for 45 s, annealing at 55°C for 45 s, and extension at 72°C for 2 min. The fragment was ligated into pET28a (+) and verified by *Eco*RI*/Hind*III digestion. The recombinant plasmid was transformed into BL21(DE3) and cultured in LB medium. The protein expression was induced with IPTG, and the cells were lysed via ultrasonication. SDS-PAGE was performed to analyze the soluble and precipitated proteins. The precipitated cells were resuspended with PBS and reacted with 1.0 g/L baicalin at 37°C for 20 h, followed by TLC and HPLC validation.

### 2.5 Minimum inhibitory concentration and fractional inhibitory concentration index

MIC assays were performed on *C. albicans* according to methods of the CLSI (formerly NCCLS) (M27-A3) (Fothergill, 2012). A single colony of *C. albicans* was picked and inoculated into YPD medium (20 g/L glucose, 10 g/L peptone, 5 g/L yeast extract powder, 14 g/L agar, pH 7.0), which was then cultured at 28°C with shaking at 200 rpm for 16 h. An appropriate amount of the culture was taken, and the cells were collected by centrifugation (3,000 × g, 5 min, 4°C) and washed three times with PBS buffer. The *C. albicans* cells were resuspended in an appropriate volume of YPD medium to achieve a cell concentration of 1 ~ 5 × 10^6^ CFU/mL. A sterile 96-well plate was taken. In well 1, only 100 μL of YPD medium was added as a blank control. Wells 3 to 12 were each inoculated with 100 μL of the aforementioned fungal suspension. In well 2, 198 μL of the fungal suspension and 2 μL of the tested drug solution were added. Wells 2 to 11 were subjected to two-fold serial dilutions, resulting in final drug concentrations of 320, 160, 80, 40, 20, 10, 5, 2.5, 1.25, and 0.625 μL/mL, respectively (baicalein as an example). The ethanol content in each well was less than 1%. Well 12 had 100 μL of fungal suspension added as a growth control. Plates were incubated at 35°C for 24 h. Optical density was measured at 630 nm, and background optical densities were subtracted from that of each well. Each of the tested drug solution was tested in triplicate. The definition of MIC_90_ was defined as the lowest concentration at which the absorbance was less than 90% of the control wells.

The fractional inhibitory concentration index (FICI) was defined as the sum of the MIC of each drug when used in combination divided by the MIC of the drug used alone. The MIC of combined drug was determined by checkerboard assay. The two combined drugs were subjected to serial dilutions in both directions (rows A-F and columns 2–11) on a 96-well plate in a two-dimensional checkerboard. First, baicalein was added to the fungal suspension that had already been adjusted to the desired concentration, and then it was added to wells 2–11 of each of the rows (rows A-F), resulting in final concentrations of baicalein of 160, 80, 40, 20, 10, and 5 μg/mL in each row, respectively. Subsequently, 2 μL of fluconazole was added to the second well of each row (rows A-F), and a two-fold serial dilution was performed to achieve final concentrations of fluconazole of 320, 160, 80, 40, 20, 10, 5, 2.5, 1.25, and 0.625 μg/mL, respectively. Rows G and H were used to determine the MIC of fluconazole and baicalein as single drug, respectively. The ethanol content in each well was less than 1%. Column 1 contained only YPD medium as a blank control, and column 12 contained only the fungal suspension as a growth control. The MIC_90_ of combined drug was defined as the lowest concentration at which the absorbance was less than 90% of the control wells. Synergism and antagonism were defined by FIC indices of <0.5 and > 4, respectively. An FIC index result of >0.5 but <4 was considered indifferent (Odds, 2003).

### 2.6 Mycelium inhibition assay

A single colony of *C. albicans* was picked and inoculated into YPD medium, which was then cultured at 28°C with shaking at 200 rpm for 16 h. An appropriate amount of the culture was taken, and the cells were collected by centrifugation (3,000 × g, 5 min, 4°C) and washed three times with PBS buffer. The *C. albicans* cells were resuspended in an appropriate volume of RPMI 1640 to achieve a cell concentration of 1 ~ 5 × 10^6^ CFU/mL. In a sterile 12-well culture plate, 1 mL of the fungal suspension was added to each well, followed by the addition of different concentrations of nystatin, fluconazole, baicalein, and baicalin. The mixtures were vortexed to ensure homogeneity and then incubated statically at 35°C for 3 h. The growth of hyphae was observed under an inverted microscope and photographed. RPMI 1640 was used as a blank control, and the fungal suspension without drugs was used as a growth control (Cao et al., 2008).

### 2.7 Evaluation of antifungal effects of transformation solution

The transformation solution was obtained after 6 days and mixed with a *C. albicans* suspension (initial concentration 1 × 10^6^ CFU/mL) at 28°C. The mixed cultures from day 2 and day 8 were diluted to appropriate concentrations, and the viable fungal counts in each group were determined using the plate colony counting method. Meanwhile, three control experiments were conducted, which included the addition of the test fungal to *L. rhamnosus* culture broth, the addition of the test fungal to MRS broth, and the addition of the test fungal to MRS broth containing baicalin. All other culture conditions and counting methods were consistent.

## 3 Results

### 3.1 Baicalein exhibited enhanced anti-*Candida albicans* activity compared to Baicalin

To evaluate the differences in antifungal efficacy between the primary bioactive components of *S. baicalensis*, baicalin and baicalein, their inhibitory effects were assessed toward *C. albicans*. Minimum inhibitory concentration (MIC) assays were conducted using both fluconazole and nystatin as positive controls. The results revealed that baicalein exhibited MIC_90_ values of 80 μg/mL and 160 μg/mL against fluconazole-sensitive and fluconazole-resistant strains of *C. albicans*, respectively. In contrast, baicalin displayed significantly higher MIC_90_ values of 512 μg/mL and 1,024 μg/mL against the same strains (Table 1). Nystatin outperformed both compounds, demonstrating the highest antifungal potency across both sensitive and resistant strains.

**Table 1:** Determination of MIC and FICI of four test substances.

MIC_90_ (μg/ml)	*C. albicans* (sensitive strain)	*C. albicans* (resistant strain)
FLC	2.3	320
NYS	2.0	8.0
B	512	1,024
BE	80.0	160
FLC + BE	-	2.5/10

FLC, Fluconazole; NYS, Nystatin; B, Baicalin; BE, Baicalein; FICI, Fractional Inhibitory Concentration Index; −, Unexposed data.

These findings indicate that baicalein possesses markedly stronger antifungal activity against *C. albicans* compared to baicalin, with a 6.4-fold greater inhibition capacity. Notably, baicalein was found to be more effective than fluconazole against resistant *C. albicans* strains. To further explore the potential for enhanced antifungal efficacy, the synergistic effects of baicalein in combination with fluconazole against resistant *C. albicans* strains were investigated.

The combination of baicalein and fluconazole produced a significant reduction in MIC values. The MIC for baicalein decreased from 160 μg/mL to 10 μg/mL, representing a 16-fold decrease, while the MIC for fluconazole dropped from 320 μg/mL to 2.5 μg/mL, an extraordinary 1,280-fold decrease. The fractional inhibitory concentration index (FICI) was calculated to be 0.07, which is well below the threshold of 0.5, confirming a highly significant synergistic interaction between baicalein and fluconazole in inhibiting resistant *C. albicans* strains.

### 3.2 Baicalein exhibits significant inhibition of *Candida albicans* mycelial growth

**Figure 1:**
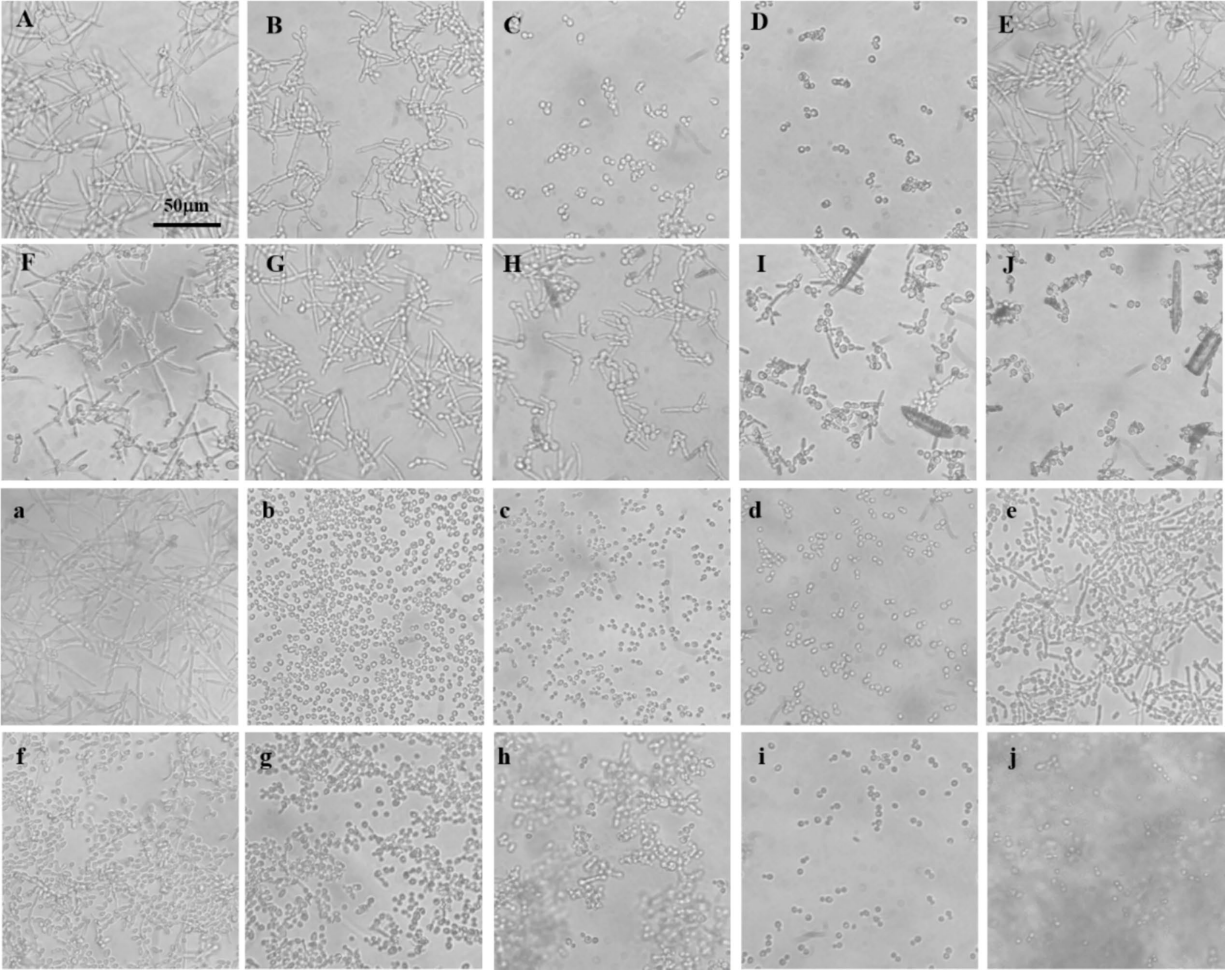
Mycelial formation in *C. albicans* sensitive and resistant strains. **(A)** No antifungal agents added; **(B)** Fluconazole at a final concentration of 16 μg/mL; **(C,D)** Nystatin at final concentrations of 16 and 32 μg/mL, respectively; **(E–G)** Baicalin at final concentrations of 128, 256, and 512 μg/mL, respectively; **(H–J)** Baicalein at final concentrations of 40, 80, and 160 μg/mL, respectively. Uppercase letters represent experiments with fluconazole-sensitive *C. albicans* strains. **(a)** no. antifungal agents added; **(b–d)** Nystatin at final concentrations of 16, 32, and 64 μg/mL, respectively; **(e–g)** Baicalin at final concentrations of 256, 512, and 1,024 μg/mL, respectively; **(h–j)** Baicalein at final concentrations of 64, 128, and 256 μg/mL, respectively. Lowercase letters represent experiments with fluconazole-resistant *C. albicans* strains.

Mycelial formation in *C. albicans* is intricately linked to biofilm development, virulence, and drug resistance. To explore the differential effects of baicalin and baicalein on mycelial inhibition, comparative experiments were conducted. The results indicated that mycelial proliferation occurred robustly in the absence of antifungal agents, while fluconazole exhibited limited inhibition of mycelial formation. In contrast, nystatin markedly suppressed mycelial growth (Figures 1A–D). Even at a concentration of 512 μg/mL, baicalin failed to inhibit mycelial formation. However, baicalein significantly reduced mycelial growth at 40 μg/mL, showing effects comparable to those observed in the fluconazole-treated group. When the baicalein concentration was increased to 160 μg/mL, the level of mycelial inhibition approached that of nystatin, displaying a concentration-dependent inhibition pattern (Figures 1E–J).

Subsequently, this analysis was extended to fluconazole-resistant *C. albicans* strains. Nystatin significantly inhibited resistant mycelial growth, with inhibition intensifying in a dose-dependent manner (Figures 1b–d). Baicalin, however, showed only partial inhibition at 256 μg/mL, and a substantial reduction in mycelial growth was observed at 1024 μg/mL (Figures 1e–g). Baicalein, by contrast, significantly inhibited the growth of resistant mycelia at a concentration of 64 μg/mL, with near-complete inhibition achieved at 256 μg/mL (Figures 1h–j).

These results demonstrate that baicalein is substantially more effective at inhibiting *C. albicans* mycelial growth than baicalin. This suggests that baicalein’s enhanced antifungal activity may be closely linked to its potent inhibition of mycelial formation, a key factor in biofilm development and drug resistance.

### 3.3 *Lactobacillus rhamnosus* efficiently converts baicalin to baicalein

Although baicalein exhibits potent anti-*C. albicans* activity and effectively inhibits mycelial formation, its low content in *S. baicalensis* limits its direct extraction and application. Baicalin, the predominant component of *S. baicalensis*, is present in quantities 80 to 100 times greater than baicalein. Therefore, utilizing microbial enzymes to hydrolyze baicalin into baicalein provides a promising approach to enhance the biological activity of *S. baicalensis* and optimize its resource utilization.

**Figure 2:**
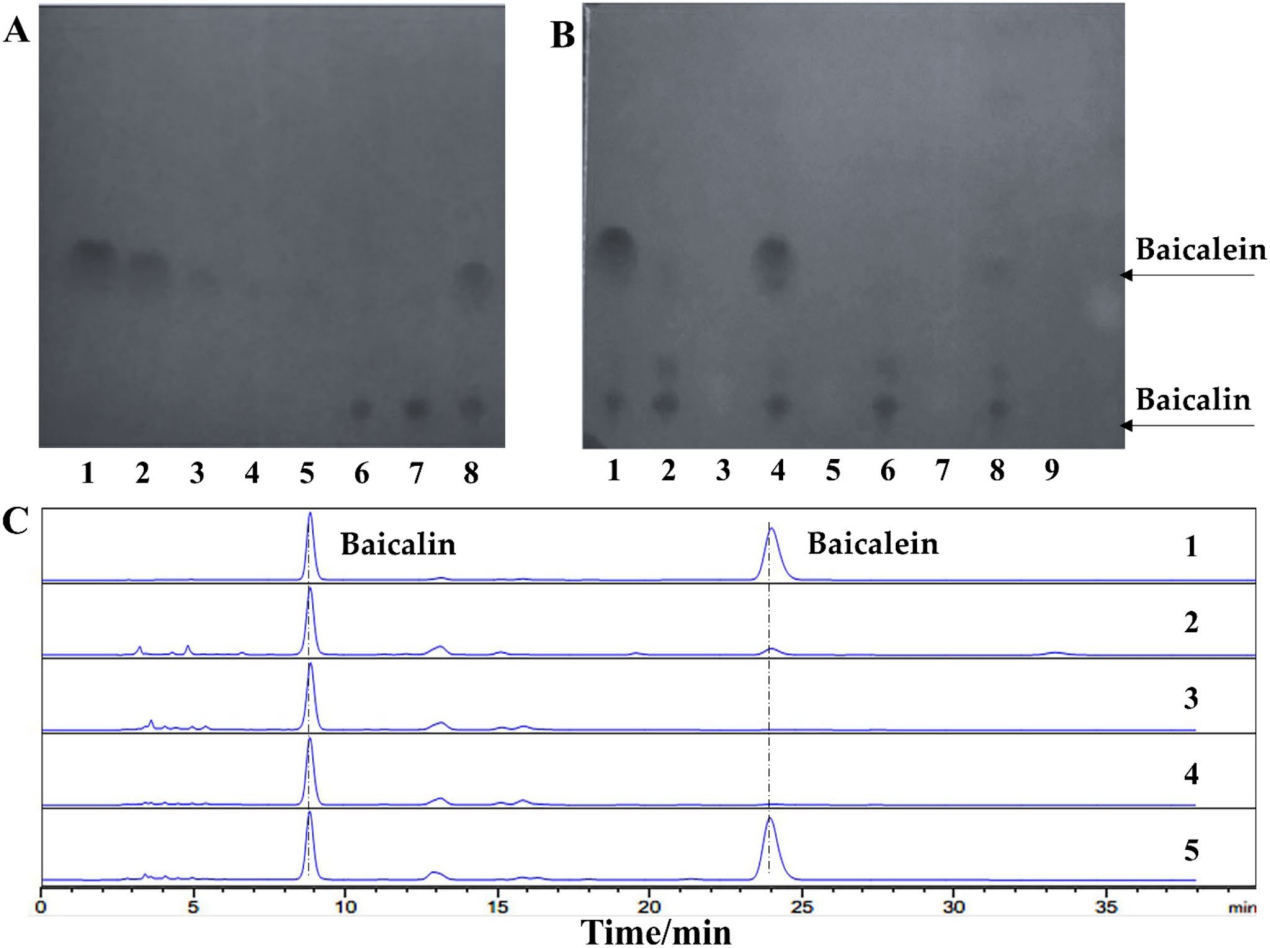
*Lactobacillus rhamnosus* efficiently converts baicalin to baicalein. **(A)** Thin-layer chromatography (TLC) analysis of baicalin and baicalein standards. Lanes 1–5: Baicalein standard samples at concentrations of 1,000, 250, 100, 50, and 25 μg/mL, respectively. Lanes 6–7: Baicalin standard samples at concentrations of 500 and 1,000 μg/mL, respectively. Lane 8: A mixture of baicalein and baicalin, each at a concentration of 500 μg/mL. **(B)** TLC analysis of baicalin conversion by four candidate strains. Lane 1: Standard mixture. Lanes 2–3: *L. paracasei* transformation solution and culture control, respectively. Lanes 4–5: *L. rhamnosus* transformation solution and culture control, respectively. Lanes 6–7: *L. gasseri* transformation solution and culture control, respectively. Lanes 8–9: *Gibberella* sp. CICC 2498 transformation solution and culture control, respectively. **(C)** High-performance liquid chromatography (HPLC) analysis of baicalin conversion by four candidate strains. Lane 1: Standard mixture. Lanes 2–5: Transformation solutions from *Gibberella* sp. CICC 2498, *L. gasseri*, *L. paracasei*, and *L. rhamnosus*, respectively.

To screen microorganisms capable of performing this transformation, thin-layer chromatography (TLC) was employed, where baicalein and baicalin exhibited distinct retention factor (Rf) values of 0.38 and 0.06, respectively. The detection limit for baicalein was 100 μg/mL (Figure 2A). Using this TLC method, 14 microbial strains were evaluated (Supplementary Figure 1), including 8 fungal species (*Gibberella* sp. CICC 2498, *Cunninghamella echinnulata* CGMCC 3.967, *Absidia coerulea* CICC 41050, *Cunninghamella blakesleeana* CGMCC 3.970, *Cunninghamella elegans* CGMCC 3.910, *Aspergillus niger*, *Penicillium*, and *Paecilomyces lilacinus*), 3 yeast strains (*Yarrowia lipolytica* CGMCC 2.1405, *Yarrowia lipolytica* CGMCC 2.2087, and *Saccharomyces cerevisiae*), and 3 strains of lactic acid bacteria (*Lactobacillus rhamnosus*, *Lactobacillus paracasei*, and *Lactobacillus gasseri*).

Preliminary screening revealed that several strains demonstrated the ability to convert baicalin (Supplementary Table 1), with *Gibberella* sp. CICC 2498, *Cunninghamella blakesleeana* CGMCC 3.970, *Aspergillus niger*, *L. paracasei*, *L. gasseri*, and *L. rhamnosus* showing varying degrees of transformation activity. However, only *Gibberella* sp. CICC 2498 and *L. rhamnosus* were able to produce detectable amounts of baicalein, with *L. paracasei* and *L. gasseri* yielding only faint baicalein spots on the TLC plate (Figure 2B). To confirm these findings, the top four candidate strains were rescreened using high-performance liquid chromatography (HPLC). The retention times of the transformation products from *Gibberella* sp. CICC 2498 and *L. rhamnosus* matched the baicalein standard. Among the tested strains, *L. rhamnosus* exhibited the largest peak area for baicalein, indicating its superior transformation efficiency (Figure 2C). Based on these results, *L. rhamnosus* were selected for further investigation into its potential as a biocatalyst for baicalin-to-baicalein conversion.

### 3.4 Optimized transformation solution exhibits enhanced inhibition of *Candida albicans*

**Figure 3:**
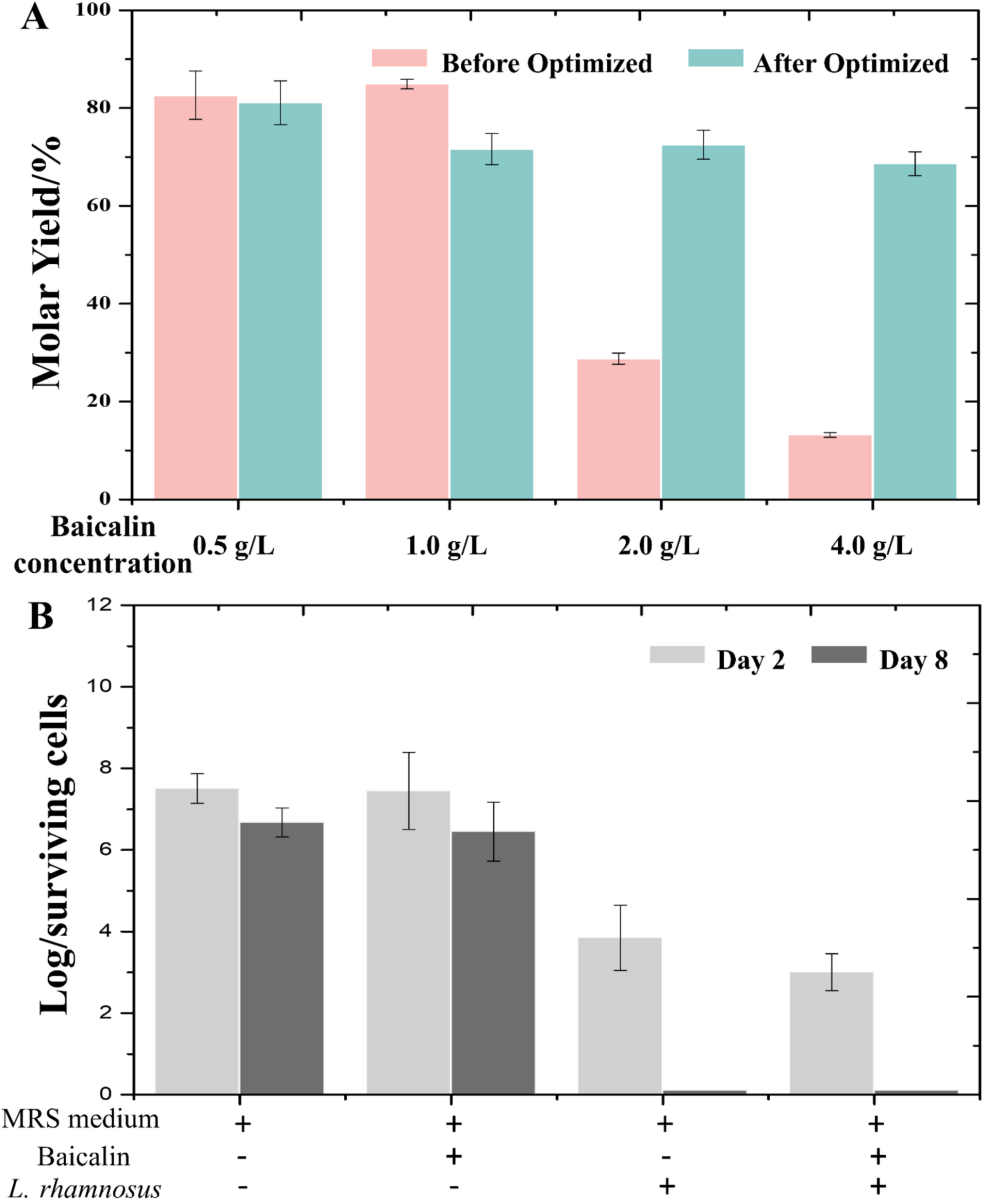
The optimized transformation solution exhibits enhanced antifungal activity. **(A)** Comparison of transformation efficiency between different substrate concentrations before and after optimization. The pink bars represent the conditions prior to optimization, while the blue bars represent the conditions post-optimization. **(B)** Inhibition of *C. albicans* by the transformation solution produced under optimized conditions at different time points (initial baicalin concentration: 2 g/L).

Given the safety profile of *L. rhamnosus* and the incomplete soluble expression of *Lr*GUS2 (Supplementary Figure 4), the potential of baicalin biotransformation using whole-cell catalysis were explored. Different concentrations of baicalin were supplemented to explore the transformation potential of *L. rhamnosus*. The results showed that the molar yield of baicalein could reach 80% at lower concentrations. However, when the concentration increased to 2 g/L and 4 g/L, the molar yields of baicalein were only 28 and 13%, respectively (Figure 3A). This indicates that *L. rhamnosus* has relatively low transformation efficiency at high concentrations of baicalin, which needs to be further improved. The efficiency of the conversion is influenced by two major factors: enzyme content and enzyme activity. Enzyme content is closely linked to the cellular growth state and the composition of the medium, while enzyme activity depends on variables such as pH, temperature, and substrate concentration.

To optimize the transformation process, a detailed single factor optimization was performed. Methanol was used as a solvent to track the reaction over time, revealing that baicalin concentration decreased while baicalein concentration increased steadily, reaching its maximum after 6 days with a molar yield of 82.5%. This suggests that after incubating the seed culture for 15 h, optimal transformation occurs over a 6-day period using actively growing cells (Supplementary Figure 2).

Subsequently, the transformation conditions were optimized by identifying the ideal carbon and nitrogen sources, as well as the culture parameters. The optimal carbon source was determined to be 10 g/L lactose, while the optimal nitrogen source was 20 g/L tryptone. Carbon sources provide energy for the growth, reproduction, and metabolic activities of microorganisms, and their degradation products can regulate enzymes in other metabolic pathways. A concentration of 10 g/L of lactose may have promoted the expression of the glycoside hydrolase in *L. rhamnosus* that is responsible for the conversion of baicalin, thereby increasing the molar yield. The lack of nitrogen atoms in the structures of baicalin and baicalein may not provide sufficient nitrogen for microbial growth. An optimal carbon-to-nitrogen ratio has been determined, for example, a C:N ratio of 1:2, which may have promoted microbial growth and thereby increased the molar yield.

The filling volume of shake flask can affect the dissolved oxygen, which in turn affects the growth of the *L. rhamnosus*. pH not only affects the growth state of the microorganisms but also has a significant impact on enzyme activity. An appropriate pH is crucial for the conversion of baicalein. Due to the relatively low solubility of baicalin in water, which reduces the conversion rate, we also compared the effects of different solvents on the conversion of baicalin. A filling volume of 20 mL, an inoculation percentage of 6% (v/v), and pH 5.0 acetic acid buffer as a solvent were found to maximize the transformation efficiency. The optimal initial pH for the culture medium was determined to be 6.5 (Supplementary Figure 2).

Combining the previously optimized single factors and validating the results across different substrate concentrations, no significant differences in transformation efficiency was observed when the substrate concentration was below 1 g/L. However, at higher substrate concentrations, the optimization led to a substantial improvement in molar yield. Under the optimized transformation conditions, the molar conversion rate of *L. rhamnosus* for a substrate concentration of 2 g/L reached 72%, which is 1.57 times higher than that before optimization. Under the optimized transformation conditions, the molar conversion rate of *L. rhamnosus* for a substrate concentration of 4 g/L reached 66%, which is 4.07 times higher than that before optimization (Figure 3A). We defined 1 unit of enzyme activity as the amount of enzyme required to produce 1 μM baicalein per day. Under optimal conditions, the activity of *β*-glucuronidase in *L. rhamnosus* was approximately 0.921 U/mg.

Probiotic colonization in the intestine plays a crucial role in improving gut flora and promoting health benefits. As *L. rhamnosus* is a well-known probiotic, it is hypothesized that its combination with baicalein could exert enhanced antimicrobial effects. To explore this possibility, a preliminary investigation was conducted into the ability of *L. rhamnosus* transformation solution, optimized at a substrate concentration of 2 g/L, to inhibit *C. albicans.*In control conditions using MRS medium, *C. albicans* exhibited normal growth, with logarithmic viable counts of 7.505 on day 2 and 6.672 on day 8. In MRS medium supplemented with baicalin, the logarithmic viable counts were 7.447 on day 2 and 6.447 on day 8, indicating that baicalin alone did not significantly inhibit fungal growth.

However, in the *L. rhamnosus* culture group, a significant reduction in *C. albicans* viable counts was observed, with a logarithmic value of 3.845 on day 2 and no detectable surviving *C. albicans* on day 8, demonstrating the strong antifungal activity of *L. rhamnosus*. Remarkably, the transformation solution group exhibited even stronger antifungal effects, with a logarithmic value of 3.000 on day 2, which was 0.845 lower than that of the *L. rhamnosus* culture group alone (Figure 3B). These results confirmed that the transformation solution possessed enhanced antifungal activity, consistent with our initial hypothesis.

### 3.5 Characterization of *Lr*GUS2 in *Lactobacillus rhamnosus*

*β*-glucuronidase (β-GUS) enzymes hydrolyze glycosidic bonds in baicalin, facilitating the conversion to baicalein. Most β-GUS enzymes belong to the glycoside hydrolase family 2 (GH2), with some members distributed across the GH1, GH30, and GH79 families. Kim et al. (2009) successfully amplified an 1812 bp sequence from *Lactobacillus brevis* RO1 (GenBank No. FJ597974), leading to the heterologous expression of *Lb*GUS2, which hydrolyzes baicalin to baicalein. The primers used for this amplification were designed based on the glucuronidase gene annotated in *L. brevis* ATCC 367.

**Figure 4:**
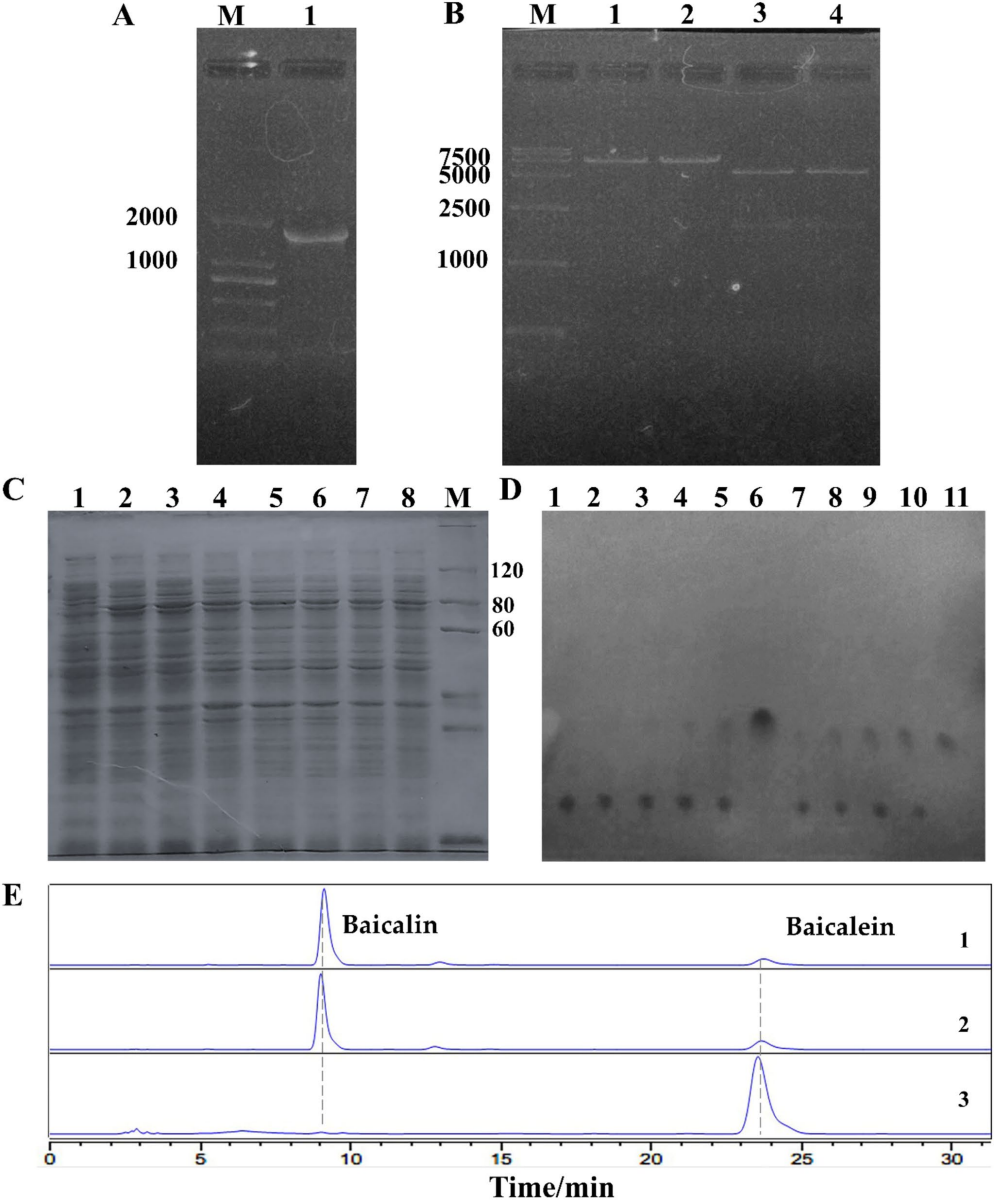
Cloning and characterization of β-glucuronidase (*Lr*GUS2) in *L. rhamnosus*. **(A)** Electropheresis of the *uidA* gene cloned from *L. rhamnosus*. **(B)** Validation of the recombinant plasmid pET-28a(+)-*uidA*. Lanes 1–2: *EcoR*I digestion yields a 7.2 kb fragment; Lanes 3–4: *EcoR*I*/Hind*III double digestion yields fragments of 5.3 kb and 1.8 kb, respectively. **(C)** Soluble expression of *Lr*GUS2 induced by different concentrations of IPTG. Lanes 1–8: IPTG concentrations of 0, 10, 20, 50, 100, 200, 500, and 1,000 μmol/L, respectively; M: PAGE-MASTER Protein Standard Plus (Genscript). **(D)** TLC validation of baicalin conversion. Lanes 1–5: BL21 (DE3) transformation time points at 0, 1, 3, 6, and 22 h; Lane 6: Baicalein standard; Lanes 7–11: BL21 (DE3)-pET-28a(+)-*uidA* transformation time points at 0, 1, 3, 6, and 22 h. **(E)** HPLC validation of baicalin conversion. Lane 1: BL21 (DE3) transformation; Lane 2: BL21 (DE3)-pET-28a(+)-*uidA* transformation without IPTG induction; Lane 3: BL21 (DE3)-pET-28a(+)-*uidA* transformation with 20 μmol/L IPTG.

In our study, a homologous gene, *uid*A (>NC_013198.1:49825–51636) was identified in the genome of *L. rhamnosus* GG ATCC 53103, annotated as β-GUS. Primers were designed based on this gene, successfully amplified an 1832 bp gene sequence in our strain (Figure 4A). Sequencing revealed 27 base mutations, all of which were silent mutations, resulting in 100% homology to the annotated *uid*A protein sequence (Supplementary Figure 3). The amplified fragment was then ligated into the pET-28a(+) vector, produced the recombinant plasmid pET-28a(+)-*uid*A. After digestion with *EcoR*I, the recombinant plasmid size was confirmed to be 7201 bp, and *EcoR*I*/Hind*III double digestion yielded fragments of 5369 bp and 1832 bp (Figure 4B), indicated successful construction of the recombinant plasmid.

The pET-28a(+)-*uid*A was transformed into *Escherichia coli* BL21 (DE3) cells, and protein expression was induced using IPTG. The expressed protein was designated *Lr*GUS2 (Protein ID: BAI40577.1). Solubility analysis was conducted with 1 mmol/L IPTG, revealing significant protein bands upon induction, though most of the expressed protein was found in the pellet, with only a small portion present in the supernatant. Optimization of the IPTG concentration indicated that soluble expression decreased with increasing IPTG concentration, with the highest soluble expression observed at 20 μmol/L IPTG (Figure 4C, Supplementary Figure 4).

Due to the low level of soluble protein expression, the whole-cell catalysis were employed to evaluate the ability of *Lr*GUS2 to convert baicalin to baicalein. BL21 (DE3) cells harboring pET-28a(+)-*uid*A were induced to convert 1 g/L baicalin. The transformation results showed a gradual increase in baicalein content over time, with baicalin being completely converted after 22 h. In contrast, BL21 (DE3) cells without the recombinant plasmid produced only trace amounts of baicalein (Figure 4D). HPLC analysis further confirmed that the host strain and BL21 (DE3)-pET-28a(+)-*uid*A without IPTG retained a large amount of baicalin. However, upon induction with 20 μmol/L IPTG, BL21 (DE3)-pET-28a(+)-*uid*A fully converted the baicalin substrate to baicalein (Figure 4E). These results demonstrate that the expression of *Lr*GUS2 significantly enhanced the conversion efficiency, further confirming its ability to hydrolyze baicalin into baicalein.

## 4 Discussion

The antifungal activity of baicalein has emerged as a potent alternative to existing therapies, particularly given the increasing resistance of *C. albicans* to conventional antifungal drugs. The results of this study demonstrate that baicalein exerts stronger antifungal effects compared to baicalin, both in sensitive and resistant strains of *C. albicans*. This finding is significant, as baicalin is present in much higher concentrations in *S. baicalensis* than baicalein, making baicalin the more accessible precursor for therapeutic use. By employing *L. rhamnosus* as a biocatalyst, an efficient method was established to convert baicalin into baicalein, overcoming the limitation of baicalein’s low natural abundance. Furthermore, the combination of baicalein with fluconazole has been shown to enhance antifungal efficacy, suggesting a promising synergistic approach for combating drug-resistant fungal infections.

Previous studies have demonstrated various enzymatic methods for baicalin transformation. Muderrisoglu (Muderrisoglu and Yesil-Celiktas, 2019) used a commercial enzyme derived from *Helix pomatia* to transform 60 ppm (0.06 g/L) baicalin with a conversion efficiency of 99%. Similarly, *Lb*GUS2, cloned and expressed from *Lactobacillus brevis* RO1, completely transformed 0.15 mM (0.07 g/L) baicalin (Kim et al., 2009). Sakurama (Sakurama et al., 2014) utilized *Lc*GUS30 to transform 0.5 mM (0.23 g/L) baicalin, while immobilized *Ec*GUS2 achieved a 77% conversion of 0.09 mM (0.04 g/L) baicalin and retained 90% activity after 26 days of storage (Jiang et al., 2007). While these enzymatic methods demonstrated high conversion rates, the substrate concentrations used in these studies were generally below 1 g/L. In contrast, the current study used *L. rhamnosus* to achieve a molar yield of 66% at a substrate concentration of 4 g/L, indicating the potential of *L. rhamnosus* as an efficient biocatalyst for baicalin transformation at higher substrate concentrations.

Yu et al. (2020) achieved a molar yield of 88.4% using purified *β*-D-glucuronidase from *Aspergillus niger* b.48, catalyzing 10 g/L baicalin. However, the pathogenic nature of *Aspergillus niger* raises safety concerns for practical applications. In contrast, using *Lactobacillus* strains, which are generally regarded as safe (GRAS), offers a safer and more sustainable approach. For example, Ku et al. (2011) used *Lactobacillus delbrueckii* Rh2 to convert 0.04 mM (0.02 g/L) baicalin with a 37.5% molar yield after 6 h at 50°C. However, the addition of 1% lactose in our study significantly enhanced baicalin transformation, resulting in a molar yield of 66%, which is higher than that reported by Xu and Ji (2013), who achieved 16.3% molar yield with *L. brevis* RO1 in a milk-based medium containing 3.5 mM (1.56 g/L) baicalin. These results suggest that *L. rhamnosus* offers a more robust and scalable approach for the green biotransformation of baicalin into baicalein.

Baicalein has been reported to exhibit strong *in vitro* antifungal activity against *Candida krusei* isolates, with a minimum inhibitory concentration (MIC) of 2.7 μg/mL. Its antifungal mechanism involves depolarizing mitochondrial membrane potential in a concentration-dependent manner, disrupting mitochondrial homeostasis, and inhibiting fungal growth (Kang et al., 2010). Furthermore, the combination of baicalein and fluconazole has been shown to significantly reduce the MIC of *C. albicans*, *C. tropicalis*, and *C. parapsilosis*, with a synergistic effect observed in *C. parapsilosis* (synergistic inhibition concentration index = 0.207) (Serpa et al., 2012). In this study, a detailed comparison was made between the activity of baicalin and baicalein against both sensitive and resistant strains of *C. albicans*. The results demonstrated that baicalein exhibited significantly stronger antifungal activity than baicalin in both strain types. Moreover, the combination of baicalein and fluconazole further enhanced the antifungal effect against resistant *C. albicans* strains, with a fractional inhibitory concentration index (FICI) of 0.07, indicating a strong synergistic effect. It is hypothesized that baicalein may inhibit the expression of efflux pumps, thereby overcoming drug resistance in *C. albicans* (Huang et al., 2008).

The formation of biofilms in *C. albicans* contributes to the inherent resistance of these pathogens to many commonly used antifungal drugs. Biofilms create a protective matrix that shields the fungal cells from antifungal agents, allowing the cells to persist even in the presence of drugs. Our study investigated the effects of baicalin and baicalein on hyphal formation, which plays a key role in biofilm development. The results showed that baicalein had a markedly stronger ability to inhibit hyphal growth than baicalin, and this inhibitory effect was dose-dependent. These findings are consistent with those of Cao et al. (2008), who reported that baicalein inhibits biofilm formation at various growth stages of *C. albicans*. The exact mechanism by which baicalein inhibits biofilm formation is not yet fully understood. However, Li et al. (2022) proposed that baicalein disrupts glycolysis by targeting the *C. albicans* Eno1 protein, potentially reducing side effects on human cells by selectively binding to CaEno1. Other potential targets for baicalein include 1,3-*β*-D-glucan synthase and glycosylphosphatidylinositol proteins (Zhou et al., 2023). These proteins are essential components of the fungal cell wall, and their inhibition could explain baicalein’s potent antifungal activity.

In terms of antimicrobial activity, probiotics have been widely studied for their inhibitory effects against various pathogens. For instance, *L. rhamnosus* MDC 9661 has been shown to inhibit the growth of *Penicillium chrysotoxum* and *Penicillium plumbum* (Bazukyan et al., 2018), while *L. rhamnosus* SCB0119 exhibited inhibitory effects against *Escherichia coli* ATCC25922 and *Staphylococcus aureus* ATCC6538 (Peng et al., 2022). Furthermore, cell-free supernatants (CFSs) from *L. rhamnosus* and *Lactobacillus plantarum* have been found to inhibit biofilm formation of *C. albicans* and *C. tropicalis*, potentially due to metabolites secreted by the bacteria (Poon and Hui, 2023). Our findings corroborate these results, showing that *L. rhamnosus* culture medium alone significantly inhibited the growth of *C. albicans*, as evidenced by a decrease in logarithmic values from 7.505 to 3.845. These results align with previous studies that found *L. rhamnosus* GG decreased the log growth of *C. albicans* isolates by an average of 1.7 to 2.1 log units (Alziadi et al., 2021).

The probiotic’s ability to inhibit fungal growth, combined with baicalein’s potent antifungal activity, offers a dual approach to treating fungal infections. Interestingly, the combined solution of *L. rhamnosus* and baicalein did not exhibit significant synergy, as evidenced by the antifungal activity not being substantially higher than either treatment alone. This lack of synergy may be due to the different mechanisms through which *L. rhamnosus* and baicalein exert their antifungal effects. Claes et al. (2012) elucidated that *L. rhamnosus* GG secretes major proteins Msp1 and Msp2, which play critical roles in fungal inhibition. Msp1, for instance, acts as a D-glutamyl-L-lysine endopeptidase, reducing hyphal formation through chitin hydrolase activity (Allonsius et al., 2019). In contrast, baicalein appears to target other critical pathways, such as glycolysis and cell wall biosynthesis, suggesting that these two agents may not work in concert in the way traditional synergistic therapies do.

In addition to the antifungal activity of *L. rhamnosus*, the biotransformation capabilities of this probiotic strain present a significant advantage for the large-scale production of baicalein. Several studies have explored the enzymatic conversion of baicalin to baicalein, focusing on enzymes such as *β*-glucuronidases from *S. baicalensis* roots, including *s*GUS79 (Sasaki et al., 2000) and *Sb*GUS79 (Zhang et al., 2005), both of which belong to the GH79 family. Xu et al. (2018) identified and characterized TpGUS79 from *Talaromyces pinophilus*, which hydrolyzes GL at a rate 38 times higher than baicalin and 1,114 times higher than pNP-*β*-GlcA. Another significant study by Sakurama et al. (2014) introduced *Lc*GUS30, an enzyme from *Lactobacillus brevis* subsp. *coagulans*, belonging to a new subfamily of GH30. *Lc*GUS30 displayed a catalytic efficiency for pNP-β-GlcA that was 19 times higher than that for baicalin, underscoring the variability in catalytic abilities across β-GUS enzymes.

Our study focused on the β-glucuronidase enzyme *Lr*GUS2, derived from *L. rhamnosus*, for the biotransformation of baicalin to baicalein. Bioinformatics analysis of *Lr*GUS2 using the Pfam database revealed that it belongs to the GH2 family, sharing structural similarities with other well-characterized β-GUS enzymes. Specifically, *Lr*GUS2 contains three conserved domains: a sugar-binding domain (residues 17–183), an immunoglobulin-like β-sandwich domain (residues 198–277), and a catalytic domain (residues 279–596). Key catalytic residues, such as Glu415 (acid–base residue), Tyr475 (active site), and Glu509 (nucleophile), were conserved in *Lr*GUS2, aligning with previously reported β-GUS enzymes, including EcGUS2 (Zhang et al., 2009) and LbGUS2 (Kim et al., 2009). Comparative analysis revealed that *Lr*GUS2 shares 43.5% identity and 56.7% similarity with *Ec*GUS2, and 40.4% identity and 54.9% similarity with *Lb*GUS2, suggesting moderate homology. Despite this moderate similarity, phylogenetic analysis positioned *Lr*GUS2 on a distinct branch within the GH2 family, indicating potential differences in its catalytic features. Notably, four conserved residue fragments 362-(FKMAAAAFLGGLNQSFF)-378,444-(RTFTL-EDDT)-453, 520-(KLPS)-524, and 584-(AAAF)-587 were identified as distinguishing conserved features of *Lr*GUS2, suggesting that it may possess unique catalytic properties compared to other β-GUS enzymes, such as *Ec*GUS2, *Lg*GUS2, and *Lb*GUS2 (Supplementary Figure 5).

Moving forward, additional studies are needed to explore the molecular mechanisms behind the antifungal activity of baicalein, particularly its interactions with fungal metabolic pathways and drug resistance mechanisms. Furthermore, the optimization of *Lr*GUS2 for industrial-scale baicalein production could significantly enhance the accessibility of this promising antifungal compound for therapeutic use. The potential for combining baicalein with other antifungal agents, such as fluconazole, also warrants further investigation, as these combination therapies could provide a powerful tool in combating antifungal resistance.

## 5 Conclusion

This study highlights the potent antifungal activity of baicalein, both as a standalone treatment and in combination with fluconazole, particularly against drug-resistant strains of *C. albicans*. Additionally, the probiotic *L. rhamnosus* demonstrated both direct antifungal activity and the ability to convert baicalin into baicalein, offering a dual-function strategy for antifungal therapy. The successful cloning and characterization of *Lr*GUS2 provides important insights into the enzymatic conversion process, which could be further optimized for industrial-scale baicalein production. These findings not only support the development of baicalein as a novel antifungal agent but also promote the sustainable utilization of *S. baicalensis* through green biotransformation technologies.

## Data Availability

The datasets presented in this study can be found in online repositories. The names of the repository/repositories and accession number(s) can be found in the article/Supplementary material.
