# Supplementary material for: Baicalein as a potent antifungal agent against Candida albicans: synergy with fluconazole and sustainable production through probiotic-mediated bioconversion
Source: Front Microbiol. 2025 Feb 25;16:1562103. doi: 10.3389/fmicb.2025.1562103 (PMC11893618; doi:10.3389/fmicb.2025.1562103)
Supplement: Supplementary file 1 [file Data_Sheet_1.docx]

Supplementary Material

## Supplementary Figures


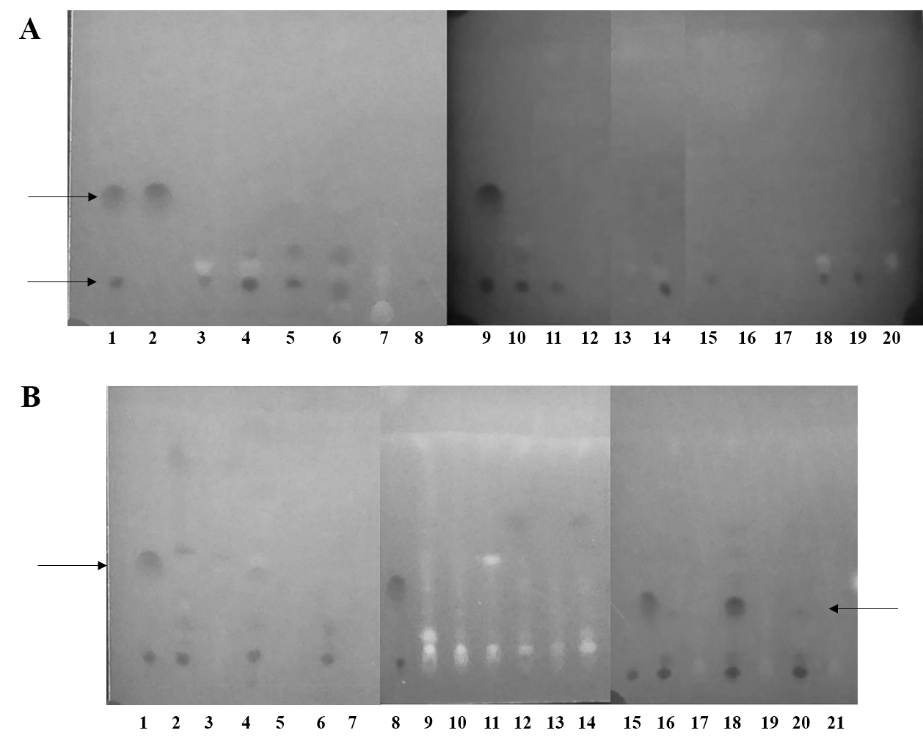


**Supplementary Figure 1.** **Analysis of Baicalin Transformation by 14 Strains**

(A)Lane 1, 9: Baicalin and baicalein standard mixture. Lane 2: Baicalein standard. Lanes 3–4: *Cunninghamella blakesleeana* 3.970 transformation. Lanes 5–6: *Aspergillus niger* transformation. Lanes 7–8: *Cunninghamella blakesleeana* 3.970 and *Aspergillus niger* culture control. Lanes 10–11: *Cunninghamella elegans* 3.910 transformation. Lane 12: Culture control. Lane 13: Culture medium control. Lane 14: Baicalin standard. Lanes 15–16: *Cunninghamella echinulata* CGMCC 3.967 transformation. Lane 17: Culture control. Lanes 18–19: *Absidia coerulea* CICC 41050 transformation. Lane 20: Culture control.

(B)Lanes 1, 8, 15: Baicalin and baicalein standard mixture. Lanes 2–3: *Paecilomyces lilacinus* transformation and culture control. Lanes 4–5: *Gibberella* sp. CICC 2498 transformation and culture control. Lanes 6–7: *Penicillium* transformation and culture control. Lanes 9–10: *Saccharomyces cerevisiae* transformation and culture control. Lanes 11–12: *Yarrowia lipolytica* CGMCC 2.1405 transformation and culture control. Lanes 13–14: *Yarrowia lipolytica* CGMCC 2.2087 transformation and culture control. Lanes 16–17: *Lactobacillus gasseri* transformation and culture control. Lanes 18–19: *Lactobacillus rhamnosus* transformation and culture control. Lanes 20–21: *Lactobacillus paracasei* transformation and culture control. The black arrows indicate the potential new products generated during transformation, while the red arrows represent baicalein.


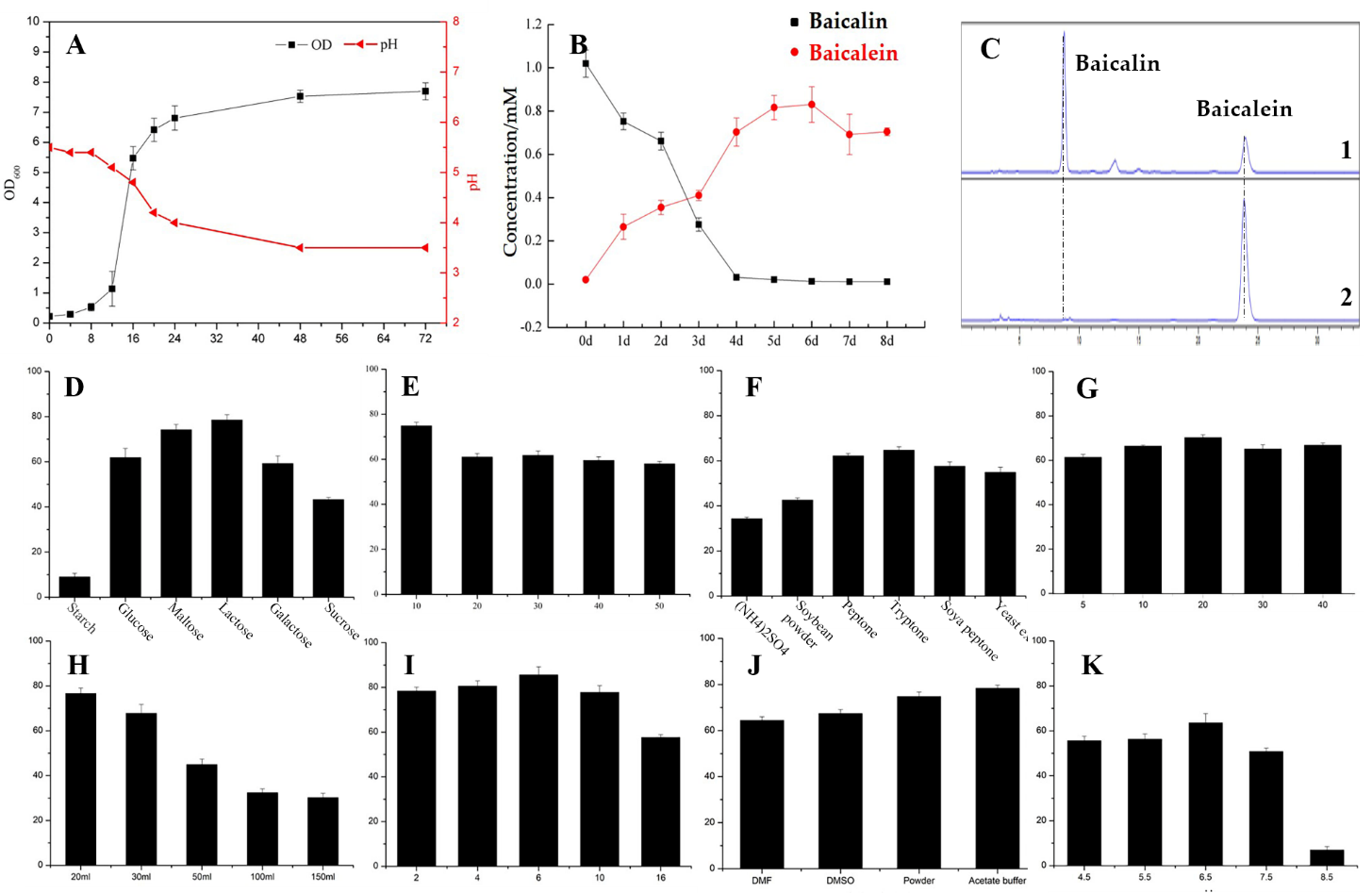


**Supplementary Figure 2. Optimization of Conditions to Improve Baicalin Transformation Efficiency by Lactobacillus rhamnosus**

(A) Growth curve of *L. rhamnosus*. (B) Dynamic transformation process of baicalin by *L. rhamnosus*. (C) HPLC analysis of baicalin conversion using different methods: 1. Resting cell conversion;2. Growth phase conversion. (D) Effect of different carbon sources on baicalin conversion efficiency. (E) Effect of various lactose concentrations on conversion efficiency. (F) Impact of different nitrogen sources on conversion efficiency. (G) Effect of varying concentrations of tryptone on conversion efficiency. (H) Effect of liquid volume loading on conversion efficiency. (I) Effect of inoculation amount on conversion efficiency. (J) Effect of different cosolvents on conversion efficiency. (K) Influence of pH on baicalin conversion efficiency.


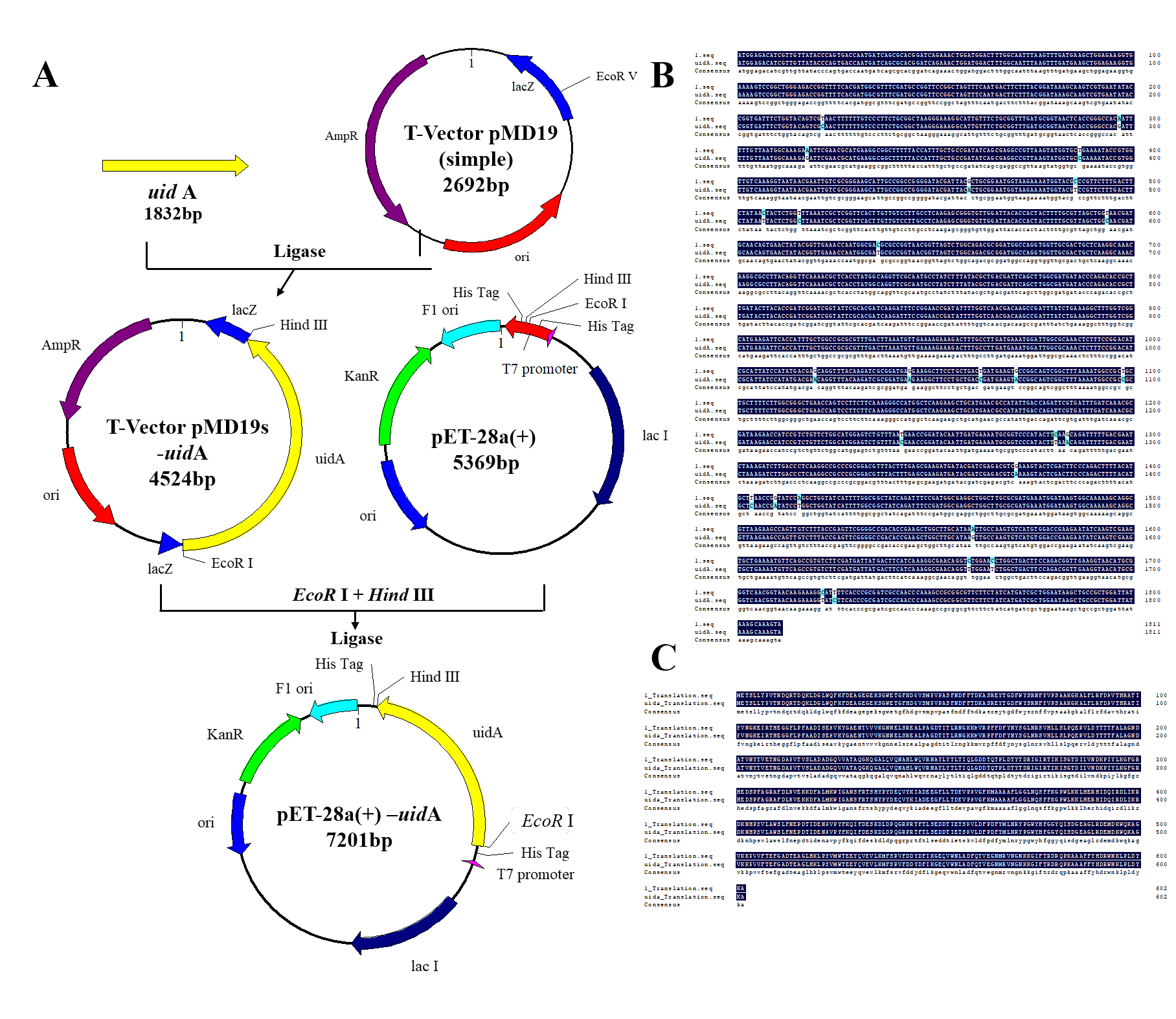


**Supplementary Figure 3. Construction and Sequence Analysis of the Recombinant Plasmid pET-28a(+)-*uid*A**

(A) Construction of the recombinant plasmid pET-28a(+)-*uid*A. (B) Nucleic acid sequence comparison of *uid*A. (C) Amino acid sequence comparison of *uid*A.


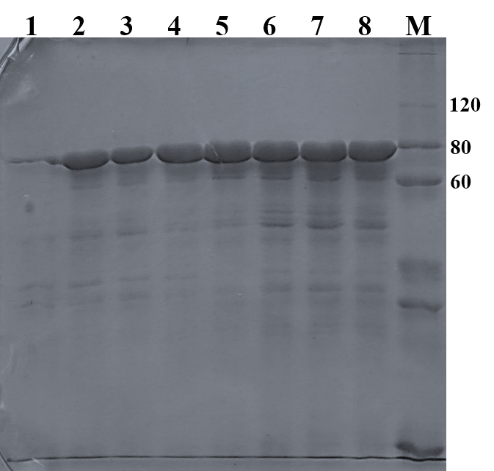


**Supplementary Figure 4. *Lr*GUS2 Precipitation Induced by Different Concentrations of IPTG**

Lanes 1–8: *Lr*GUS2 precipitation induced by IPTG at concentrations of 0, 10, 20, 50, 100, 200, 500, and 1000 μmol/L, respectively. M: PAGE-MASTER Protein Standard Plus (Genscript).


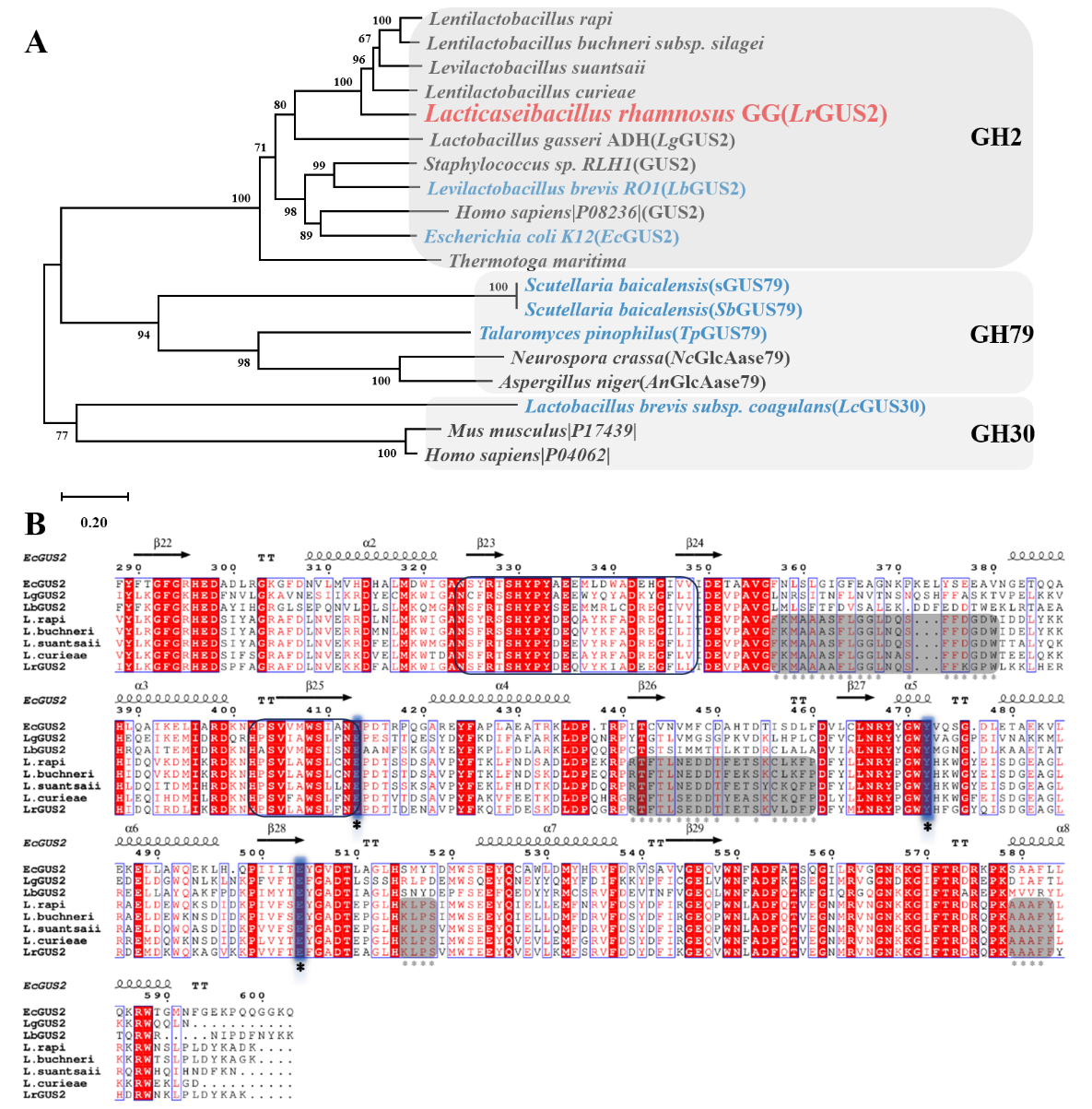


**Supplementary Figure 5. Phylogenetic and Multi-Sequence Analysis of *Lr*GUS2**

(A)Phylogenetic analysis of *Lr*GUS2 with other β-glucuronidases (GUSs) from the GH2, GH79, and GH30 families, based on their amino acid sequences. Enzymes shown in blue font belong to different families and have the ability to hydrolyze baicalin, while enzymes in red font were characterized in this study. The phylogenetic tree was constructed using the neighbor-joining method in MEGA 11. The GH2 GUSs include enzymes from *L. rapi* (A0A512PRA4), *L. buchneri* subsp. *silagei* (J9W2U8), *L. suantsaii* (A0A4Q0VJB5), *L. curieae* (A0A1S6QH27), *L. gasseri* (AAK07836.1), *Staphylococcus* sp. RLH1 (Q9AFA2), *L. brevis* (ACU21612.1), *Homo sapiens* (P08236), *E. coli* K12 (P05804), and *T. maritima* (Q9X0F2). The GH79 GUSs include enzymes from *S. baicalensis* (Q9LRC8 and BAA97804.1), *T. pinophilus* (GAM42629.1), *N. crassa* (Q7SFB0), and *A. niger* (A2QEQ6). The GH30 GUSs include enzymes from *L. brevis* subsp. *coagulans* (BAO73305.1), *Mus musculus* (P17439), and *Homo sapiens* (P04062).

(B)Multi-sequence alignment of GH2 GUSs, including *Ec*GUS2 from *E. coli* K12, *Lg*GUS2 from *L. gasseri*, *Lb*GUS2 from *L. brevis*, and GUS2 from *L. rapi*, *L. buchneri* subsp. *silagei,* *L. suantsaii*, and *L. curieae*. Dashed boxes highlight the conserved sequences containing catalytic residues, and stars indicate the catalytic residues. The dark gray background highlights the unique motif of *Lr*GUS2.

## Supplementary Tables

**Table S1. Ability of Baicalin Transformation.**

| **Microorganism** | **Ability*** |
| --- | --- |
| *Gibberella sp.*CICC 2498 | ++ |
| *Cunninghamella echinulata* CGMCC 3.967 | D |
| *Absidia coerulea* CICC 41050 | + |
| *Cunninghamella blakesleeana* 3.970 | + |
| *Cunninghamella elegans* 3.910 | + |
| *Aspergillus niger* | + |
| *Penicillium* | + |
| *Paecilomyces lilacinus* | + |
| *Yarrowia lipolytica* CGMCC 2.1405 | D |
| *Yarrowia lipolytica* CGMCC 2.2087 | D |
| *Saccharomyces cerevisiae* | D |
| *Lactobacillus rhamnosus* | +++ |
| *Lactobacillus paracasei* | ++ |
| *Lactobacillus gasseri* | ++ |

(D) No product observed;
(+) More than 80% of the substrate remains;
(++) 50–80% of the substrate remains;
(+++) Less than 50% of the substrate remains (determined via HPLC detection at λ = 280 nm).
